# Role of Epithelial-Mesenchymal Transition in Pancreatic Ductal Adenocarcinoma: Is Tumor Budding the Missing Link?

**DOI:** 10.3389/fonc.2013.00221

**Published:** 2013-09-17

**Authors:** Eva Karamitopoulou

**Affiliations:** ^1^Clinical Pathology Division, Institute of Pathology, University of Bern, Bern, Switzerland; ^2^Translational Research Unit, Institute of Pathology, University of Bern, Bern, Switzerland

**Keywords:** pancreatic cancer, epithelial-mesenchymal transition, tumor budding, prognosis, biomarker

## Abstract

Pancreatic ductal adenocarcinoma (PDAC) ranks as the fourth commonest cause of cancer death while its incidence is increasing worldwide. For all stages, survival at 5 years is<5%. The lethal nature of pancreatic cancer is attributed to its high metastatic potential to the lymphatic system and distant organs. Lack of effective therapeutic options contributes to the high mortality rates of PDAC. Recent evidence suggests that epithelial-mesenchymal transition (EMT) plays an important role to the disease progression and development of drug resistance in PDAC. Tumor budding is thought to reflect the process of EMT which allows neoplastic epithelial cells to acquire a mesenchymal phenotype thus increasing their capacity for migration and invasion and help them become resistant to apoptotic signals. In a recent study by our own group the presence and prognostic significance of tumor budding in PDAC were investigated and an association between high-grade budding and aggressive clinicopathological features of the tumors as well as worse outcome of the patients was found. The identification of EMT phenotypic targets may help identifying new molecules so that future therapeutic strategies directed specifically against them could potentially have an impact on drug resistance and invasiveness and hence improve the prognosis of PDAC patients. The aim of this short review is to present an insight on the morphological and molecular aspects of EMT and on the factors that are involved in the induction of EMT in PDAC.

## Pancreatic Cancer

Pancreatic ductal adenocarcinoma (PDAC) is a common cancer with dismal prognosis (1) that escapes early detection and resists treatment (2). Most patients have advanced stage disease at presentation with a median survival of less than 1 year (1, 3). Surgical resection is the only potentially curative treatment of PDAC (3). Classical histomorphological features like tumor size, blood vessel, or lymphatic invasion, and presence of lymph node metastases constitute essential prognostic determinants in pancreatic cancer and are invariably included in the pathology reports, with tumor stage being the most important of all (3). The lethal nature of PDAC has been attributed to the propensity of PDAC cells to rapidly disseminate to the lymphatic system and distant organs (4). However, even patients with completely resected, node-negative PDACs eventually die of their disease. Within this context and considering the fact that the management of PDAC remains suboptimal and that adjuvant therapy has resulted to limited progress, the identification of additional reliable and reproducible prognostic markers that would enable better patient stratification and eventually provide a guide toward a more successful and individualized therapy, is mandatory (1, 5).

## Epithelial-Mesenchymal Transition

Epithelial-mesenchymal transition is a biologic process that allows epithelial cells to undergo the biochemical changes that enable them to acquire a mesenchymal phenotype, including enhanced migratory capacity, invasiveness, elevated resistance to apoptosis, and increased production of extracellular matrix (ECM) components (6, 7). EMT is characterized by loss of cell adhesion, down regulation of E-cadherin expression, acquisition of mesenchymal markers (including N-cadherin, Vimentin, and Fibronectin), and increased cell motility (6). Both EMT and mesenchymal-epithelial transition (MET), the reversion of EMT, are essential for developmental and repair processes like implantation, embryo formation, and organ development as well as wound healing, tissue regeneration, and organ fibrosis (8). However, EMT also occurs in neoplastic cells that have undergone genetic and epigenetic changes. These changes affect both oncogenes and tumor suppressor genes that enable cancer cells to invade and metastasize. Moreover, some neoplastic cells may go through EMT retaining many of their epithelial properties while other cells are becoming fully mesenchymal (9).

Many molecular processes are involved in the initiation of EMT including activation of transcription factors, expression of specific cell-surface proteins, reorganization and expression of cytoskeletal proteins, production of ECM-degrading enzymes, and changes in the expression of specific microRNAs (miRNAS). The above factors can also be used as biomarkers to detect cells in EMT state (10). EMT has been linked to cellular self-renewal programs of cancer stem cells and apoptosis-anoikis resistance, which are features of therapeutic resistance (11).

The zinc finger transcription factors Snail, Slug, Zeb1, and Twist repress genes responsible for the epithelial phenotype and represent important regulators of EMT (6, 7, 12). In PDAC Snail expression has been reported to be seen in nearly 80% of the cases and Slug expression in 50% (13). Snail expression was inversely correlated with E-cadherin expression and decreased E-cadherin expression was associated with higher tumor grade. Similarly, poorly differentiated pancreatic cancer cell lines showed higher levels of Snail and lower levels of E-cadherin compared with moderately differentiated cell lines (13) while silencing of Zeb1 leaded to up-regulation of E-cadherin and restoration of an epithelial phenotype (14). Zeb1 expression in PDAC also correlated with advanced tumor grade and worse outcomes (14–16) and was shown to be primarily responsible for the acquisition of an EMT phenotype, along with increased migration and invasion in response to NF-κB signaling in pancreatic cancer cells (16).

## EMT and Tumor Budding

Tumor budding reflects a type of diffusely infiltrative growth consisting of detached tumor cells or small cell clusters of up to five cells at the invasive front of gastrointestinal carcinomas (17–22). Tumor buds represent a non-proliferating, non-apoptotic, highly aggressive subpopulation of tumor cells that display migratory and invasive capacities (23). The aim of tumor buds seems to be the invasion of the peritumoral connective tissue, the avoidance of the host’s defense and finally the infiltration of the lymphatic and blood vessels with the consequence of local and distant metastasis. The EMT process by allowing a polarized cell to assume a more mesenchymal phenotype with increased migratory capacity, invasiveness, and resistance to apoptosis seems to play a major role in the development of tumor buds. In fact, tumor buds are thought to result from the process of EMT. Thus, although formally tumor budding cannot be equated with EMT, several similarities between the two processes, including activation in WNT signaling, can be shown (24). The detachment of tumor buds from the main tumor body is accomplished by loss of membranous expression of the adhesion molecule E-cadherin. Activation of WNT signaling is further suggested by nuclear expression of b-catenin in tumor-budding cells, as well as increase of laminin 5 gamma 2 and activation of Slug and Zeb1 (24, 25).

The presence of high-grade tumor budding has been consistently associated with negative clinicopathologic parameters in gastrointestinal tumors (26–30). In a previous study from our group we could show that tumor budding occurs frequently in pancreatic cancer and is a strong, independent, and reproducible, highly unfavorable prognostic factor that may be used as a parameter of tumor aggressiveness and as an indicator of unfavorable outcome, even within this group of patients with generally poor prognosis. Moreover, tumor budding was proven to have a more powerful prognostic ability than other more classic prognostic factors including TNM stage, thus adding relevant and independent prognostic information (31).

## EMT and miRNAs

MicroRNAS are small non-coding RNAs of 18–25 nucleotides, excised from 60 to 110 nucleotide RNA precursor structures (32). MiRNAs are involved in crucial biological processes, including development, differentiation, apoptosis, and proliferation, through imperfect pairing with target messenger RNAs of protein-coding genes and the transcriptional or post-transcriptional regulation of their expression (33, 34).

Recent studies illustrate the role of miRNAs on the regulation of gene expression and proteins in metastasis. For example, it has been shown that miR-10b, which is up-regulated by EMT transcription factor Twist, is associated with increased invasiveness and metastatic potential (35, 36). Furthermore, it was shown that the miR-200 family (miR-200a, miR-200b, miR-200c, miR-141, and miR-429) and miR-205 play critical roles in regulating EMT by directly targeting the mRNAs encoding E-cadherin repressors Zeb1 and Zeb2 (37). Moreover, recent studies showed that members of the miR-200 family by inducing EMT can regulate the sensitivity to epidermal growth factor receptor (EGFR) in bladder cancer cells and to gemcitabine in pancreatic cancer cells (38). Conversely, Zeb1 represses the transcription of miR-200 genes by directly binding to their promoter region, thereby forming a double-negative feedback loop (39). On the other hand, miR-200 family can also promote the conversion of mesenchymal cells to epithelial-like cells (MET) suggesting that these miRNAs may also favor metastatic outgrowth.

Recent studies aiming at the evaluation of miRNAs in pancreatic cancer have shown that specific miRNAs are dysregulated in PDAC while the higher expression of some miRNA species was able to distinguish between benign and malignant pancreatic tissue (40). For example, miR-21 was shown to be overexpressed in 79% of pancreatic cancers as opposed to 27% of chronic pancreatitis (41). In resected PDAC specimens high levels of miR-200c expression strongly correlated with E-cadherin levels and were associated with significantly better survival rates compared with patients whose tumors had low levels of miR-200c expression (42).

## Chemoresistance and EMT

Cells undergoing EMT become invasive and develop resistance to chemotherapeutic agents. Moreover, EMT can be induced by chemotherapeutic agents, and stress conditions such as exposure to radiation or hypoxia (43, 44). Up-regulation of Twist has been shown to be associated with resistance to paclitaxel in nasopharyngeal, bladder, ovarian, and prostate cancers (45). In colorectal cancer cell lines, chronic exposure to oxaliplatin leaded to the development of the ability to migrate and invade with phenotypic changes resembling EMT (spindle-cell shape, loss of polarity, intercellular separation, and pseudopodia formation) by the oxaliplatin-resistant cells (46).

Pancreatic cancer remains today an extremely lethal disease largely because of its resistance to existing treatments (47). EMT has been shown to contribute significantly to chemoresistance in several cancers, including pancreatic cancer (30, 48, 49). Induction of gemcitabine resistance in previously sensitive cell lines resulted in development of an EMT phenotype and was associated with an increased migratory and invasive ability compared to gemcitabine sensitive cells (49). Moreover, gene expression profiling of chemoresistant cells showed a strong association between expression of the EMT transcription factors Zeb1, Snail, and Twist and decreased expression of E-cadherin (39, 50). Silencing of Zeb1 with siRNA resulted to MET (51) and restored chemosensitivity (14). Interestingly, maintenance of chemoresistance in cell lines that have undergone EMT is dependent on Notch and NF-κB signaling (30). Inhibition of Notch-2 down regulates Zeb1, Snail, and Slug expression, attenuates NF-κB signaling, and reduces the migratory and invasive capacity of the gemcitabine resistant cells (30).

Epithelial-mesenchymal transition can also confer resistance to targeted agents. For example, lung cancer cell lines that have undergone EMT, became resistant to the growth inhibitory effects of EGFR kinase inhibition (erlotinib) *in vitro* and in xenografts (47) as well as other EGFR inhibitors such as gefitinib and cetuximab (48) Thus, EMT can lead to resistance to multiple agents and result to rapid progression of the tumor. Clarifying the correlation between EMT and drug resistance may help clinicians select an optimal treatment.

## Conclusion

Pancreatic cancer remains an extremely lethal disease partly because of the poor response to existing treatments. Accumulating evidence suggests that EMT plays an important role in PDAC progression, is associated with stem cell features of the PDAC cells and seems to significantly contribute to the chemoresistance of pancreatic cancer. Moreover, is associated with more aggressive tumor characteristics and with poor patient survival. Because of its role in therapy response and tumor progression, targeting EMT could potentially reduce drug resistance and have a great impact in the survival of PDAC patients.

**Figure 1:**
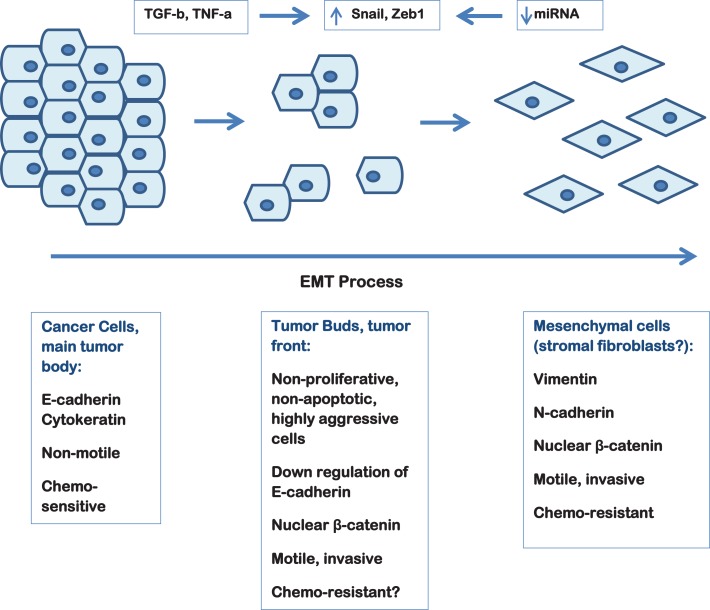
**Schematic representation of the EMT process in pancreatic cancer depicting the hypothetical link to tumor budding**.

Tumor budding thought to be the result of the EMT process is commonly observed in PDAC and high-grade tumor budding has been proven to have an independent adverse prognostic impact in the survival of PDAC patients. Figure 1 depicts tumor budding as a possible transition between a fully epithelial and a fully mesenchymal phenotype of the tumor cells in PDAC. Moreover, cancer cells in tumor buds have been shown to have EMT and cancer stem cell characteristics. The further characterization of the budding cells at a protein and gene level in order to identify a “molecular budding-promoting profile” will lead to a better understanding of the tumor-stroma interaction at the area of the invasive front and help to further elucidate the similarities between budding cells, EMT process and cancer stem cells in pancreatic cancer.

Investigating these issues will allow us to gain further insight into pancreatic carcinogenesis, and provide us with a platform on which to build future studies leading to the identification of new therapeutic interventions.

## Conflict of Interest Statement

The author declares that the research was conducted in the absence of any commercial or financial relationships that could be construed as a potential conflict of interest.
